# EP10 Hyperinflammatory syndrome in a patient with rheumatoid arthritis and COVID-19

**DOI:** 10.1093/rap/rkaa052.009

**Published:** 2020-11-03

**Authors:** Melissa Ong, Mark Gibson, Gerald Coakley

**Affiliations:** Rheumatology Department, Lewisham and Greenwich NHS Trust, London, United Kingdom

## Abstract

**Case report - Introduction:**

Severe acute respiratory coronavirus 2 (SARS-CoV-2) is a novel virus that can lead to an excessive immune activation and cytokine response known as Coronavirus disease 2019 (COVID-19) which predominantly affects the lungs. Patients with chronic inflammatory disease on biological immunosuppressive treatments may be at a higher risk of contracting SARS-CoV-2. However, it is yet to be determined whether immunomodulatory medications used in inflammatory diseases have protective capabilities against severe outcomes.

**Case report - Case description:**

A 51-year old female with a 13-year history of rheumatoid arthritis (RA) presented to hospital with fever, exertional breathlessness, and a non-productive cough. She was diagnosed with seropositive erosive RA at the age of 38 and was on 6-monthly Rituximab infusions and Leflunomide on admission. She had relatively stable pulmonary fibrosis (diagnosed in 2010). Her chest CTs in 2010 and 2018 noted bilateral basal subpleural ground glass change with limited honeycombing and spirometry study revealed FEV1 of 2.2 (82% predicted), VC of 2.7 (87% predicted), DLCO of 7.0 (78% predicted) and kCO of 1.6 (78% predicted).

On admission in March 2020, she was hypoxic (oxygen saturation of 88% in room air) and had raised inflammatory markers (CRP 341mg/dL, d-Dimer 914ng/ml, Ferritin 3141ng/ml, LDH 672U/L). Her last Rituximab infusion was 3 months prior and leflunomide was withheld on admission. SARS-CoV-2 PCR nasopharyngeal swab was positive, and she was recruited to the RECOVERY trial, being randomized to Lopinavir-Ritonavir for 10 days. Her oxygen requirements increased, and a CT pulmonary angiogram excluded pulmonary embolism but revealed ground glass changes and extensive multilobar consolidation. She was eligible for recruitment into RECOVERY-2 (tocilizumab) given the ongoing oxygen requirement and elevated CRP, but she was randomised to usual care. She was commenced on 80mg of IV methylprednisolone, a dose chosen because of its proven effectiveness in Acute Respiratory Distress Syndrome. She clinically improved and was discharged from hospital 20 days after starting Methylprednisolone with a CRP of 17mg/dL.

Two months after discharge, the patient had repeat spirometry study which noted FEV1 of 1.4 (57% predicted), VC of 1.5 (52% predicted), DLCO of 2.4 (28% predicted) and kCO of 1.0 (47% predicted). A repeat high-resolution chest CT reported significant improvement of peripheral ground glass changes and consolidation, but she is still fatigued and more breathless than previously.

**Case report - Discussion:**

The RECOVERY trial concluded that Dexamethasone reduced mortality in intubated patients and in hospitalised patients with COVID-19 with a high oxygen requirement. The results were published after this patient was discharged.

A hyperinflammatory response to COVID-19 is seen in a subset of patients, and our own hospital data suggest that this condition affects around 5% of admitted COVID-19 patients, but that extreme hyperferritinaemia above 10,000 is extremely rare. Similar responses (known as Haemophagocytic Lymphohistiocytosis [HLH]) are seen with a variety of viral and bacterial infections, in malignancy and in inflammatory rheumatic diseases (Macrophage Activation Syndrome [MAS]), but typically HLH and MAS patients have ferritin > 10,000.

It appears unlikely that true HLH is a significant manifestation of COVID-19 infection, but moderate hyperferritinaemia is not uncommon and the results of this study, taken together with case reports and series from China and Italy suggest that similar treatments to those used in HLH may transform the prognosis for COVID-19 patients in this subset.

It is unknown whether the recent Rituximab infusion had a role in reducing the “cytokine storm” and delaying progression to severe COVID-19. However, it may be argued that the remaining T cells in B cell depleted patients are sufficient for viral clearance.

The long-term impact of SARS-CoV-2 on pulmonary function is still unclear. Our patient had a major deterioration in her lung function when compared to her baseline. There was severe reduction in gas transfer post COVID-19. However, her repeat high resolution CT chest reported substantial improvement in ground glass changes and consolidation. The long-term prognosis is still uncertain.

Initial fears that patients on DMARDs and biological therapies for inflammatory rheumatic disease would be extremely vulnerable to COVID-19 have not been confirmed, but patients with extra-articular manifestations on combinations of DMARDs and biological therapies may be a subset at higher risk.

**Case report - Key learning points:**

Our Intensivist colleagues, early in the COVID-19 outbreak, were understandably cautious about using heavily immunosuppressive treatments for a life-threatening viral infection. Using a multi-disciplinary approach at a time when knowledge of how to treat this condition was rudimentary, along with informed consent from an intelligent and thoughtful patient, we were able to plot a middle path to suppress hyperinflammation without using massively immunosuppressive doses of steroid, with a successful outcome.

This patient illustrates one aspect of the hyper-inflammatory response seen in a subset of the most critically ill patients with COVID-19. At the time of writing, the RECOVERY 2 trial is yet to be published, but the rapid improvement in inflammatory markers including CRP and Ferritin, along with a dramatic improvement in clinical state, suggest that relatively modest doses of parenteral steroid have life-saving potential at far lower cost and greater worldwide availability than biological therapies such as Tocilizumab or Anakinra.

Trials of Tocilizumab in RECOVERY2 and of Anakinra coordinated by the Hyperinflammation Histio UK Haemophagocytosis Across Specialty Collaboration (HASC), as well as international randomised controlled trials will be critical in determining the optimal treatment strategy for this subset of critically ill COVID-19 patients. The experience of our patient suggests that one arm of such studies should include a relatively modest dose of parenteral steroid, be that Dexamethasone or Methylprednisolone, particularly given that COVID-19 is affecting countries across the developing, as well as the developed, world.

